# Skeletal Muscle Uncoupling Proteins in Mice Models of Obesity

**DOI:** 10.3390/metabo12030259

**Published:** 2022-03-17

**Authors:** Lidija Križančić Bombek, Maša Čater

**Affiliations:** Institute of Physiology, Faculty of Medicine, University of Maribor, 2000 Maribor, Slovenia; masa.cater@um.si

**Keywords:** uncoupling protein, skeletal muscle, insulin, diabetes, obesity

## Abstract

Obesity and accompanying type 2 diabetes are among major and increasing worldwide problems that occur fundamentally due to excessive energy intake during its expenditure. Endotherms continuously consume a certain amount of energy to maintain core body temperature via thermogenic processes, mainly in brown adipose tissue and skeletal muscle. Skeletal muscle glucose utilization and heat production are significant and directly linked to body glucose homeostasis at rest, and especially during physical activity. However, this glucose balance is impaired in diabetic and obese states in humans and mice, and manifests as glucose resistance and altered muscle cell metabolism. Uncoupling proteins have a significant role in converting electrochemical energy into thermal energy without ATP generation. Different homologs of uncoupling proteins were identified, and their roles were linked to antioxidative activity and boosting glucose and lipid metabolism. From this perspective, uncoupling proteins were studied in correlation to the pathogenesis of diabetes and obesity and their possible treatments. Mice were extensively used as model organisms to study the physiology and pathophysiology of energy homeostasis. However, we should be aware of interstrain differences in mice models of obesity regarding thermogenesis and insulin resistance in skeletal muscles. Therefore, in this review, we gathered up-to-date knowledge on skeletal muscle uncoupling proteins and their effect on insulin sensitivity in mouse models of obesity and diabetes.

## 1. Introduction

Thermogenesis is of utmost importance for maintaining a stable body temperature of around 36–37 °C in humans [1] and around 36–38 °C in different strains of mice [2,3]. This temperature homeostasis is ensured by shivering and nonshivering thermogenesis, mainly in the brown adipose tissue (BAT) and skeletal muscle. In healthy adult humans, BAT is scarce and becomes dysfunctional or even proinflammatory in obese individuals favoring the development of type 2 diabetes [4]. In contrast, skeletal muscle shivering and nonshivering thermogenesis remain active throughout adult life. However, BAT and skeletal muscle thermogenesis remain substantial throughout their lives in mice. 

Parallel to its role in heat production, the skeletal muscle, being the largest glucose sink in the body, largely contributes to glucose homeostasis. Glucose enters skeletal muscle cells through type 4 glucose transporters (GLUT4), which translocate to the plasma membrane in response to increased blood glucose and insulin levels, thus allowing massive entry of glucose into cells. These processes are enhanced under increased energy demands such as physical activity or cold exposure. Since glucose is also a fuel for heat production, it is of paramount importance that its transport into cells is adequate, and that skeletal muscle cells respond properly to insulin signaling. In diabetes, however, skeletal muscle cells are resistant to insulin signaling, and glucose entry into the cytoplasm is impaired. Consequently, less glucose is available for cellular metabolism, thus affecting thermogenic processes [5]. Furthermore, the derailed glucose metabolism also results in impaired lipid and protein metabolism and their regulation. 

This review first briefly describes the metabolic and physiological mechanisms of shivering and nonshivering thermogenesis in skeletal muscle. Then, we focus on mitochondrial coupling and uncoupling processes and their interaction. We emphasize tissue specificity in expressing different isoforms of uncoupling proteins (UCPs) and their roles. In the next section, we focus on insulin secretion from pancreatic beta cells, insulin resistance, and the functioning of skeletal muscle UCPs in mouse models of obesity and diabetes. We also discuss gender and age differences in UCP expression and their correlation with adulthood weight gain. The second part of the article reviews studies of obesity and diabetes mouse models, as well as transgenic and knockout genetic alterations. Lastly, we give a brief overview of diet-induced obesity and diabetic mouse models, including the effects of caloric restriction diets that are very promising in diabetes management. 

## 2. Metabolic and Physiological Mechanisms of Shivering and Nonshivering Thermogenesis in Skeletal Muscle

In cold exposure, total heat production in the body can increase by up to five times that of the resting metabolic rate at room temperature. The major source of metabolic heat production used for conserving body temperature is skeletal muscle tissue. It dissipates heat in shivering and nonshivering thermogenic processes, which together can contribute around 40% of heat production in cold exposure [6,7].

### 2.1. Shivering Thermogenesis

Shivering thermogenesis in skeletal muscle is driven by neural mechanisms involved in recruiting the muscles into the shivering response and regulating the substrates used to fuel the metabolic processes. The research shows that the preoptic area of the hypothalamus is the main thermoregulatory center [8]. It receives sensory input from thermoreceptors in the skin, thermoreceptors in the vicinity of internal organs [9], and somatosensory fibers in the dorsal spinal horn [10], as well as from the brain and spinal cord neurons sensitive to thermal stimuli [7,8]. The central neural network responsible for the recruitment of shivering integrates the input information. It sends feedback to thermoregulatory effectors such as skin vasculature, muscle spindles, and BAT to initiate the shivering response [11,12]. In humans, muscle-shivering thermogenesis was estimated to account for up to 40% of whole-body energy expenditure during a mild cold exposure [6]. To produce heat by oxidation, the shivering muscle uses different combinations of carbohydrates, lipids, and proteins [13]. For sustained shivering for hours, carbohydrate reserves in the form of glycogen and selective recruitment of type II muscle fibers are vital [14,15]. Haman et al. reported that in the case of low carbohydrate stores in human muscles, lipids were the predominant fuel for shivering thermogenesis, with 53% of total heat produced. Of the remaining total heat production, 28% originated from carbohydrates and 19% from proteins. Conversely, in the case of high carbohydrate stores, cold-induced muscle shivering used 23% lipids, 65% carbohydrates, and 12% proteins for heat production. Regardless of glycogen stores, plasma glucose oxidation remained a minor fuel source, accounting for 7–13% of the total heat production [15].

### 2.2. Nonshivering Thermogenesis

The two greatest nonshivering contributors to heat production in skeletal muscle are unequivocally the uncoupling of the mitochondrial oxidative phosphorylation by UCPs, also called the proton leak [7,16], and uncoupling of the sarco-endoplasmic reticulum Ca^2+^-ATPase (SERCA) pump, also called futile calcium cycling [17,18]. In addition, energy-releasing cellular processes involving enzymes such as myosin ATPase and creatine kinase also dissipate some heat, thus contributing to thermogenesis. Since mitochondrial uncoupling is extensively described in the following sections, we only briefly present other nonshivering processes in this section.

#### 2.2.1. Sarco-Endoplasmic Reticulum Ca^2+^-ATPase (SERCA) Pump

The proper functioning of muscle cells depends on maintaining ionic gradients across cell membranes. It is achieved by ATPases pumping ions against their concentration gradients using energy from the hydrolysis of adenosine triphosphate (ATP). Muscle contraction is triggered by an increase in sarcoplasmic Ca^2+^ concentration due to the opening of dihydropyridine receptors on the sarcoplasmic reticulum (SR), releasing Ca^2+^ from the SR lumen into the sarcoplasm. Calcium ions bind to myofilaments setting into motion the myosin–actin cross-bridge cycling and mitochondrial oxidative metabolism, which subsequently activates SERCA and ensures that in optimally coupled conditions, two Ca^2+^ ions are pumped back into the SR lumen at the expense of hydrolyzing one molecule of ATP [19,20]. However, in a nonideal live setting, the byproduct of the imperfectly coupled Ca^2+^ transport-to-ATP hydrolysis is heat [21], accounting for up to 40–50% of the murine fast- and slow-twitch muscles’ resting metabolic rate, which is equivalent to 8–10% of the total body metabolic rate [22]. In skeletal muscle, isoform SERCA 1 is expressed in fast-twitch muscle fibers. In contrast, slow-twitch muscle fibers express SERCA 1 and SERCA 2a [23], and only SERCA 1 can modulate the amount of heat produced during the ATP hydrolysis in the range of 7–32 Kcal/mol, depending on the established transmembrane Ca^2+^ gradient across the SR membrane [24]. An important regulator of SERCA’s activity is sarcolipin (SLN) [25]. In Sln −/− knockout mice, hypothermia ensued after exposure to acute cold (4 °C), since these animals could not maintain body temperature. However, overexpression of Sln in the Sln −/− mice restored muscle thermogenesis, supporting the idea that Sln is involved in SERCA-based heat production, and that its absence may result in diet-induced obesity during increased caloric intake [17]. However, SLN is not just a SERCA uncoupler, but in mice, also regulates temperature homeostasis by affecting heat production, whole-body metabolism, and weight gain. Its expression is upregulated in cases of increased metabolic demand such as different muscle diseases, exercise, cold exposure, and diet-induced obesity [26].

#### 2.2.2. Myosin ATPase

In the resting muscle, a low metabolic rate is a consequence of the transition of the myosin ATPase into a super-relaxed state, thereby slowing down its metabolism and decreasing energy consumption. At lower temperatures, the exchange of GTP for ATP on myosin ATPase and the increase in myosin phosphorylation both decrease the fraction of myosin ATPases in the super-relaxed state, thus increasing heat production and muscle energy consumption [27]. In addition, the fraction of super-relaxed myosin ATPases at rest is lower in type IIa (fast-twitch) muscle fibers, making them greater glucose and energy consumers compared to type I (slow-twitch) muscle fibers [28]. The transition of only 20% of myosin ATPases from the super-relaxed into a normal-relaxed state approximately doubles muscle thermogenesis [29]. Targeting the super-relaxed state of myosin ATPases may provide new approaches to treat obesity, high blood sugar, or type II diabetes by increasing muscle glucose utilization [30].

#### 2.2.3. Creatine Kinase

Creatine kinase catalyzes the reversible reaction of creatine phosphorylation and exists in at least four isoforms, two of which are cytosolic and two mitochondrial. Their expression is tissue-specific and compartmentalized within cells [31,32]. Despite substantial creatine metabolism in skeletal muscle, its turnover is still higher in adipose tissue [33]; therefore, it was studied mainly in this context in the past decades. In thermogenic fat (BAT and beige adipose tissue), creatine enhanced mitochondrial respiration and energy dissipation. In mice, cold exposure stimulated creatine kinase activity and induced the expression of genes linked to creatine metabolism. This induction was further enhanced in the case of absent UCP1-dependent thermogenesis, linking a futile cycle of creatine metabolism to energy expenditure and thermoregulation [34,35,36]. Upon ablation of creatine kinase B, mice showed reduced blood glucose levels, triglycerides, and leptin, as well as disrupted thermogenic capacity and glucose homeostasis [37,38]. Recently, a mechanism combining creatine thermogenesis with futile Ca^2+^ cycling in the ER has been proposed. Still, this area needs further research [35].

### 2.3. Cold Acclimation

The effects of environmental temperature on mitochondrial efficiency and ATP production can influence thermal tolerance and performance of the body. Thus, organisms developed the ability to alter mitochondrial processes through acclimation to mitigate these effects [39]. Nonshivering thermogenesis, which is used to generate heat and warm up the body, is a plastic process and is affected by environmental factors such as chronic cold [40]. The capacity for heat production increases with cold acclimation, resulting in successful coping with chronic cold stress in endothermic mammals. Interestingly, small mammals acclimate to cold differently than larger mammals.

In small mammals, sustained cold exposure causes an increased nonshivering thermogenesis [41,42,43,44,45] based on the activity of UCP1, primarily taking place in the BAT. Nonshivering BAT thermogenesis is based on norepinephrine acting on BAT adrenergic receptors. Sympathetic activation of BAT stimulates intracellular lipolysis and the production of UCP1. A release of free fatty acids (FFAs) fuels the respiratory chain in which UCP1 dissipates the mitochondrial proton gradient as heat [41,42]. UCP1 uncouples oxidative phosphorylation from ATP production and permits protons to leak back into the mitochondrial matrix from the inner-membrane space, resulting in a high rate of substrate oxidation, liberating heat in the absence of ATP synthesis. Adaptive BAT thermogenesis is sufficient to compensate for heat loss and maintaining body temperature of mammals below 10 kg. Uncoupled respiration in BAT, driven by UCP1 in the majority as well as UCP3 [46], is supported by additional heat production from ATP turnover in BAT and other tissues. Moreover, cold acclimation has been observed to enlarge BAT mass and increase BAT citrate synthase activity in mice [43]. BAT mass can be increased by hypertrophy and hyperplasia [47,48], and it counts as one of the mechanisms to increase the capacity of nonshivering thermogenesis in cold-acclimated rodents. In contrast, shivering thermogenesis does not increase in cold-acclimated small mammals [44].

In larger mammals such as humans, BAT tissue is present in much smaller volumes, therefore nonshivering BAT thermogenesis is not sufficient for maintaining body temperature on its own. Skeletal muscle plays an important role as a tissue primarily responsible for thermogenesis in mammals heavier than 10 kg. UCPs, other than UCP1, have been found to be important for their activity in skeletal muscle. UCP3 localizes in skeletal muscle and also in BAT, where its abundance is highly correlated with that of UCP1 in BAT, and plays a major role in FFA oxidation [46]. UCP4 and UCP5 reside in skeletal muscle and are also involved in FFA metabolism [49]. Acclimation to cold causes an increased basal metabolic rate to survive in cold climates. Basal metabolism changes (increase of up to 35%) and an elevated total energy expenditure have been found in arctic human populations [42]. Adaptive changes in muscle properties in response to thermogenesis occur in cooperation with BAT activity to successfully maintain metabolic homeostasis. Metabolic changes in skeletal muscles, such as increased aerobic performance due to sustained cold exposure, resemble those observed following endurance exercise training [50,51].

## 3. Coupling, Uncoupling, and Their Interplay in Skeletal Muscle Cells

**Figure 1:**
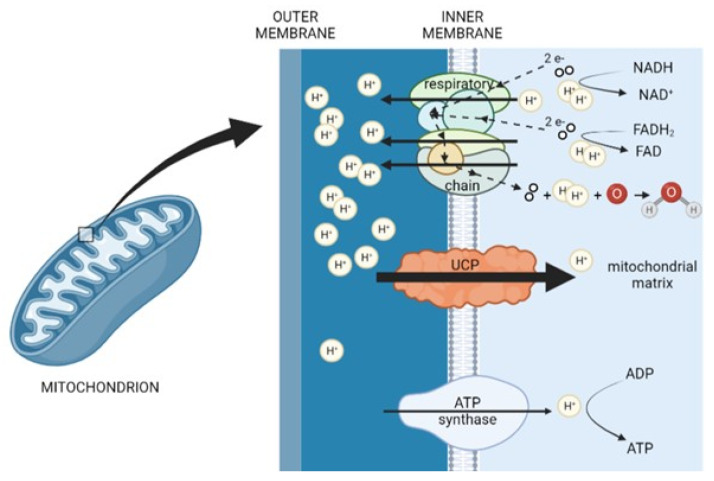
A schematic overview of UCP function and localization in a mitochondrion (created with BioRender.com, (accessed on 19 January 2022)).

In 1961, Peter Mitchell proposed the mechanism of ATP production in mitochondria known as the chemiosmotic mechanism of ATP synthesis [52,53,54], for which he received the Nobel Prize for Chemistry in 1978 [55]. According to Mitchell’s chemiosmotic theory, ATP synthesis exploits the electrochemical gradient across the inner mitochondrial membrane. This gradient arises from passing electrons from NADH and FADH_2_ formed in the Krebs cycle during the mitochondrial metabolism of energy-rich molecules through a series of membrane-bound protein complexes I–IV. At the same time, hydrogen ions (H^+^) are pumped from the mitochondrial matrix to the interspace between the mitochondrial inner and outer membrane through transmembrane complexes I, III, and IV [56,57]. Accumulation of H^+^ ions in the intermembrane space results in a powerful proton gradient across the membrane, driving their diffusion back into the matrix through F_1_F_0_ ATP synthase (Figure 1).

During this diffusion, the released energy subsequently drives phosphorylation of ADP to ATP [58,59]. Recently, Nath showed that the molecular mechanism of uncoupling the proton transport from ATP synthesis by dinitrophenols cannot be explained by simple proton conduction through uncouplers, as postulated by Mitchell’s theory. Instead, it requires a two-ion theory of energy coupling/uncoupling in ATP synthase [60]. The enzymatic activity of ATP synthase also catalyzes the reverse reaction; namely, the ATP hydrolysis, thereby influencing the forward reaction equilibrium. The reverse-forward activity of ATP-synthase is strictly controlled to prevent ATP hydrolysis at the site. This control is achieved by inhibiting the reversal of ATP synthase. An inhibitor protein named IF1 has been identified to play a role in this task, but it is insufficient by itself, and other potential regulating mechanisms are under investigation [61,62]. Due to many unresolved questions, researchers recently proposed an update of the chemiosmotic theory questioning the form of proton-motive force across the membrane based on many discoveries with novel and improved research methods [57,63].

To complicate things further, the coupling of respiration to ATP synthesis is imperfect, and mitochondrial energy consumption persists even when ATP synthesis is inhibited, confirming the presence of uncoupling or leak mechanisms [64,65,66]. In the light of strict control over ATP production, specific cellular mechanisms dissociate mitochondrial membrane potential generation from its usage to generate ATP. These processes might have evolved to control ATP production and match it to cellular consumption, or to convert electrochemical energy into thermal energy to regulate body temperature. Such bypassing of the ATP synthase through specific proteins called UCPs produces thermal energy without ATP synthesis. One of the first UCPs to be discovered and described was thermogenin, found in BAT [67,68]. Many different homologs followed in human and other taxonomic species, which we will briefly summarize in the next section [69].

In skeletal muscle, the described proton leaks through UCPs can amount to up to 20–50% of the resting metabolic rate [65], which confirms that skeletal muscle cells have an enormous potential to elevate oxygen consumption. Furthermore, in diet-induced adaptive thermogenesis, the skeletal muscle contributes 35–67% of energy expenditure [70,71]. Taken together, uncoupling of oxidative phosphorylation represents a potential target for the treatment of hyperglycemia and insulin resistance, and can be of great importance in obese and diabetic patients. 

## 4. Uncoupling Proteins

Not all UCPs are involved in thermogenesis. Although they are tissue-specific, some of the homologs colocalize but differ in their role greatly. While UCP1 is involved in nonshivering thermogenic processes in BAT in many animal taxonomic groups, UCP2 and UCP3, its close relatives, do not directly affect thermoregulation in vertebrates [72]. Both UCP2 and UCP3 can be found in adipose tissue and are linked to insulin secretion, yet their role is contradictory. Moreover, some UCPs are neuroprotective and reside in the central nervous system. Interestingly, skeletal muscle mitochondria contain the most diverse spectrum of UCP homologs—four out of five—making this tissue extremely important for studying UCPs’ function and pathologies.

### 4.1. UCP Homologs and Their Roles

**Figure 2:**
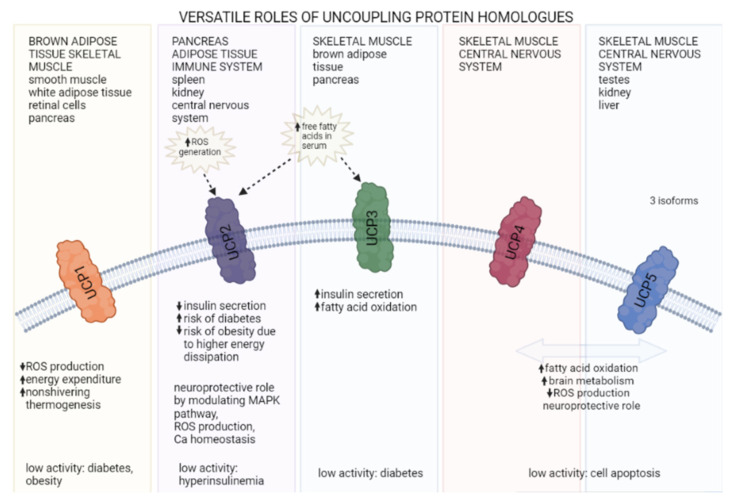
UCP homologs are present in different tissues and have distinct roles. Arrows up represent an increased activity, arrows down represent a decreased activity (created with BioRender.com, (accessed on 19 January 2022)).

The role of UCPs in the pathogenesis of diabetes mellitus has recently become a popular topic, since five homologs have been found in mammals [73,74]. Their structure is similar, but their distribution in different tissues varies greatly [75]. The physiological functions of UCPs have been studied intensively in the last three decades, yet they are still not completely elucidated. They are known for their antioxidative activity [76,77] and as glucose and lipid metabolism enhancers or regulators. Several gene polymorphisms of UCP1, UCP2, and UCP3 have been found in human diabetic and obese individuals, linking them to the development of glucose metabolism and insulin signaling pathologies [75] (Figure 2). In contrast to UCP1, which can represent as much as 10% of proteins in the inner mitochondrial membrane [45], UCP2 and UCP3 usually comprise less than 0.1% of the membrane protein content. They need specific activation for their proton transporting function [78].

#### 4.1.1. UCP1

Studies in rodents have shown that BAT starts to develop in the interscapular region during embryonic days E15–16, and that UCP1 mRNA expression increases around days E18–19 just before birth. The BAT continues to develop postnatally until between postnatal days P15–21 and remains present throughout adult life [79,80]. Recent research revealed the existence of two subpopulations of brown adipocytes in mice. One subpopulation has high thermogenic activity and high UCP1 expression, and the other has low thermogenic activity and low UCP1 expression [81]. At birth, all adipocytes express high levels of UCP1 and have high thermogenic activity to meet newborns’ thermal requirements. Postnatally, some adipocytes begin to convert to the subpopulation with low UCP1 expression so that both subpopulations coexist in adult mice and might switch between each other during normal thermogenesis at room temperature. When exposed to cold, the transcription of genes in the subpopulation with the low UCP1 expression increases, thereby increasing the total thermogenic capacity of BAT [82]. During long-term cold exposure, de novo adipogenesis was observed in BAT [83,84]. In senescence, the capacity of adipocytes to increase UCP1 expression after cold exposure becomes impaired [82].

UCP1 mainly localizes to the inner mitochondrial membrane of BAT. Its proton conductance increases in elevated concentrations of long-chain free fatty acids (FFAs) [41] and is controlled by insulin [85,86]. Apart from BAT, recent studies also reported UCP1 expression in white adipose tissue, skeletal muscle, longitudinal smooth muscle layers, retinal cells, and Langerhans islet cells [75,87].

In skeletal muscle mitochondria, the expression of UCP1 reaches only 13% of the expression in BAT and increases the GDP-sensitive proton leak [88]. The roles of UCP1 are decreasing membrane potential, reducing reactive oxygen species (ROS) generation, increasing energy expenditure, and increasing nonshivering thermogenesis [89,90,91]. Compared to BAT, the ability of UCP1 in skeletal muscle to increase glutathione levels and reduce ROS production is far greater, suggesting different specific roles and possibly distinct mechanisms of UCP1 in both tissues [88]. Some research shows that diabetes and obesity development involves specific polymorphisms of the *Ucp1* gene [92]. Mutations in *Ucp1* affect the activity or expression of the UCP1 protein and reduce regulated or basal energy expenditure, resulting in altered pancreatic function and insulin secretion [93,94].

#### 4.1.2. UCP2

UCP2 mRNA is expressed in many tissues, such as muscle, spleen, pancreas, kidney, central nervous system, and immune system. The UCP2 gene is already expressed during fetal life in murine skeletal muscle. Its expression increases immediately after birth, reaching a maximum on day 2, and steadily declines after that regardless of the lactating mother’s diet [95].

UCP2 is most widely present and highly expressed among UCPs in diabetic pancreatic beta-cells [96]; therefore, its involvement in diabetes development has been proposed. Its role in the pancreas as a negative regulator of insulin secretion has been studied intensively in ob/ob mice. The activation of UCP2 by ROS causes mitochondrial membrane proton leak, which reduces ATP synthesis in pancreatic β-cells and downregulates glucose-stimulated insulin secretion [97,98,99]. The ob/ob mice lacking UCP2 have increased ATP synthesis and glucose-stimulated insulin secretion from beta-cells in Langerhans islets [96,100]. DeSouza et al. (2007) used an antisense oligonucleotide to *Ucp2* in ob/ob mice and Swiss mice with hyperlipidemic diet-induced obesity and diabetes to inhibit UCP2 expression, resulting in metabolic improvement [99]. Finally, results from a human study on ethnicity differences in UCP2 polymorphisms demonstrated that in Asians, the UCP2-866G/A polymorphism is protective against, while the UCP2 Ala55Val polymorphism is susceptible to, type 2 diabetes [101]. Similar traits might also exist in mice, but these have not been thoroughly researched yet. 

One of the reported other roles of UCP2 is controlling immune cell activation by modulating MAPK pathways and mitochondrial ROS production [102,103]. Additionally, a neuroprotective role has been proposed. By regulating mitochondrial membrane potential, production of ROS, and calcium homeostasis, UCP2 modulates neuronal activity and inhibits cellular damage [104].

#### 4.1.3. UCP3

UCP3 is expressed in skeletal muscle and BAT [105,106,107,108]. In BAT, UCP3 is almost one order of magnitude more abundant than in skeletal muscle or heart, and is directly correlated with the abundance of UCP1 [46]. The predominant isoform in skeletal muscle is UCP3, and its expression is highly skeletal-muscle-specific [109]. In mice, UCP3 mRNA levels were highest in skeletal muscle, followed by heart, white adipose tissue, and spleen, which was somewhat different than in rats, where the expression in tissues other than skeletal muscle was negligible [110].

UCP3 expression was almost undetectable in murine muscle tissue during fetal life. In contrast, its expression became noticeable soon after birth in response to suckling and lipid intake, and steadily increased for 15 days. Interestingly, after 15 days of life, the UCP3 mRNA levels became dependent on dietary interventions. If lactating mice were fed regular high-carbohydrate chow, UCP3 expression levels in pups started to decrease, whereas if mothers were fed a high-fat diet, the levels of UCP3 expression in pups remained high [95]. Research shows that nutritional factors regulate UCP3 expression. Specifically, its expression is induced by elevated circulating FFAs, which is typical for fasting or starvation [95,111]. Pedraza et al. reported that the UCP3 expression in skeletal muscle is dramatically downregulated in lactating mice, and this effect is reversed with weaning. These changes come hand-in-hand with changes in circulating FFAs, which are reduced during lactation and return to normal after weaning [112].

Pancreatic beta cells also express UCP3 [113], linking its role to energy expenditure, glucose metabolism, diabetes, and obesity [114,115]. Pancreatic UCP3 also affects insulin secretion, but acts differently than UCP2 [113]. In humans, the expression of the *Ucp3* gene in skeletal muscle and pancreas of diabetic patients is decreased [116], suggesting *Ucp3* involvement in the development of type 2 diabetes. Muscle UCP3 is also important in FFA metabolism. It protects mitochondria from oxidative stress induced by lipids and modulates insulin sensitivity [117], making it a potential player in type 2 diabetes development. UCP3 protein levels are upregulated when FFAs’ supply to the mitochondria exceeds their oxidative capacity, and downregulated when oxidative capacity is improved.

The degradation of both UCP2 and UCP3 is very rapid [118], making their half-lives only approximately 30 min [119]. In comparison, the half-life of UCP1 is around 30 h [120]. The short half-lives of UCP2 and UCP3 enable rapid adjustments of their protein levels, which are needed when facing the rapidly changing metabolic needs and different rates of ROS production during mitochondrial oxidative processes. Because of this rapid degradation, the UCP2 protein level can decrease before the level of its mRNA drops [121]. It is crucial to consider this when evaluating data and drawing conclusions solely on mRNA expression.

#### 4.1.4. Other UCPs

UCP4 and UCP5 are mainly expressed in the central nervous system, where they play roles in brain metabolism and thermoregulatory heat production and are therefore often named neuronal UCPs [122,123]. However, their expression has also been determined in skeletal muscle, controlling energy expenditure and lipid oxidation. UCP5 is expressed in human skeletal muscle in three different isoforms, with UCP5L being the most abundant isoform, followed by UCP5S and UCP5SI [49]. UCP4 and UCP5 have a similar role in the protection against oxidative stress and mitochondrial dysfunction as other homologs [124]. High levels of UCP5 mRNA have been detected in testes, and lower levels in the kidneys and liver [49].

### 4.2. Sex Differences in UCP Expression

Studies with rodents of both genders have shown significant sex-associated differences in the regulation of UCPs, which occur due to sex hormones and other distinct gender-based biological functions [125]. Sex hormone receptors are localized in the mitochondria of specific cells and can affect mitochondrial physiology [126]. In rodents, sex hormones influence different features of skeletal muscle, such as fiber diameter and myosin heavy-chain expression [127]. They also regulate UCP1 expression in brown adipocytes [128,129].

Age plays a vital role in the sex dimorphism of UCP expression. In prepubertal age in mice, UCPs are expressed at similar levels in both sexes, with significant differences, especially in UCP1 and UCP3 expression, being observed only later in adulthood. Expression of these proteins decreased with time in adult males, while in females, UCP1 and UCP3 expression decreased during young adulthood and increased later [130]. This age-dependent UCPs expression pattern correlates with weight gain. In several studies, weight gain with aging was more significant in males than in female mice, which showed only a slight increase in body weight with senescence. This finding suggests that upregulation of UCP1 and UCP3 in BAT helps female mice avoid triglyceride accumulation in skeletal muscle and prevents obesity development [114,130,131]. 

Caloric diet feeding causes different overweight-induced expression of UCP3 in muscle and UCP1 in BAT in males than in females. Females tend to have a higher capacity to store fat when food is in excess than males, resulting in weight gain [125]. On the other hand, experiments with fasting showed interesting sex-dependent differences in UCP expression. Bazhan et al. (2019) studied sex asymmetry in the fasting effects on the transcription of the *Ucp3* gene in muscle. A significant upregulation of muscle *Ucp3* occurred in females after fasting for 24 h, while these changes were much less evident in males [132].

## 5. UCP2 and Insulin Secretion in Pancreatic Beta-Cells

The mitochondria were estimated to produce 98% of the cell’s ATP in oxidative metabolism [133]. Glucose influx into pancreatic beta-cells followed by an increase in glucose metabolism elevates cytosolic adenosine triphosphate (ATP) concentration, closing ATP-dependent K+ (KATP) channels and decreasing K+ efflux. Consequent cell membrane depolarization opens voltage-dependent Ca2+ (VDCC) channels and Ca2+ ions influx, which triggers insulin secretion [134,135,136]. The predominant UCP in beta-cells is UCP2, which regulates cellular oxidative stress, ROS production, and energy metabolism, and protects mice from aging [137,138].

Recently, a new role of UCP2 was revealed, linking its expression to the regulation of embryonic development of the pancreas. Broche et al. showed that UCP2 regulates embryonic development of the pancreas through the ROS-AKT signaling pathway by decreasing ROS production. Their study confirmed an increased pancreas size with a higher number of α- and β-cells in Ucp2−/− fetuses, a faster perinatal proliferation of endocrine cells, and increased ROS production [137].

Increased UCP2 expression can cause a lack of glucose effect on insulin secretion in type 2 diabetes [139]. UCP2 allows H+ ions to bypass ATP synthase, reducing cellular ATP content [140]. Subsequently, beta-cell membrane depolarization and glucose-stimulated insulin secretion (GSIS) decrease. A high-fat diet (HFD) or long-term exposure of beta-cells to elevated concentrations of FFAs creates glucolipotoxic conditions, which upregulate UCP2 mRNA levels and protein content up to twofold [141]. Consistent with the proposed mechanism of UCPs action, the decreased cellular ATP-to-ADP ratio suppressed glucose-induced depolarization of the plasma membrane. Furthermore, the GSIS decrease was inversely proportional to UCP2 expression [142,143]. UCP2 mRNA content increased following exposure to palmitate or its nonoxidizable derivative bromopalmitate. This observation suggests that FFAs can directly upregulate UCP2 gene expression without their preceding metabolism [141]. In contrast, UCP2 knockout (UCP2−/−) mice on a control diet have similar fasting blood glucose, fed plasma-free FFAs, and triglyceride (TG) levels as their wild-type (WT) littermates. On the other hand, their fasting blood insulin concentration was significantly higher, responding differently to the HFD [144]. In UCP2−/− mice, fasting FFA concentration following the HFD was not elevated. In addition, the fed plasma TG increase was nearly twofold less than WT control, suggesting that faster mitochondrial FFA oxidation in UCP2−/− islets prevents lipotoxicity-associated TG accumulation. Furthermore, fasting and fed plasma insulin levels following the HFD rose significantly in UCP−/− and control mice, but the increase was much higher in the UCP2−/− group. Pancreatic insulin content increased around fourfold in UCP2−/− mice, while it slightly decreased in the WT control. Interestingly, in UCP2−/− mice, the fasting blood glucose concentration was not affected by the HFD, and the fed blood glucose level increase was significantly lower than in the WT control. Further research on islets from UCP2−/− mice on a control diet showed increased insulin content per islet, relative beta-cell area per pancreas, and average islet size, but a comparable number of islets per mm2 of pancreas compared to the WT islets [144]. Following the HFD, the islet size and beta-cell area increase in UCP−/− mice were significantly larger than in WT islets. In addition, the number of islets per mm2 of the pancreas increased significantly, which was not evident in control islets [144].

When researching mitochondrial metabolism, Joseph et al. showed that basal insulin secretion in WT and UCP−/− mice on the HFD was elevated; however, GSIS was attenuated in WT mice, while UCP−/− mice showed significantly increased GSIS. The beta-cells from UCP−/− islets had no alterations in mitochondrial membrane potential, ATP/ADP ratio, and cytosolic Ca^2+^ responses to high glucose concentration after palmitate treatment compared to the dysfunctional responses of WT cells. The HFD neither resulted in glucose sensitivity loss nor elevated TG concentration as it did in the WT control, because the palmitate oxidation was faster in the UCP2−/− islets [145]. These data suggest that UCP2−/− beta-cells can resist the toxic effects of a high-lipid environment by preventing TG accumulation due to faster FFA oxidation rates and maintaining highly functional glucose-dependent metabolism–secretion coupling [145].

The most studied obesity mouse models, the ob/ob mice, have elevated UCP2 expression and impaired insulin secretion [96,99,144]. While in lean mice, GSIS improved after a short-term knockdown of islet UCP2, it remained unaffected in ob/ob mice, confirming the dysfunctionality of glucose homeostasis in the ob/ob diabetes model. In addition, this loss of glucose homeostasis and insulin secretion impairment were preceded by an increase in UCP2 expression [146].

## 6. UCPs in Mouse Models of Diabetes and Obesity

### 6.1. Obesity and Diabetic Models

**Figure 3:**
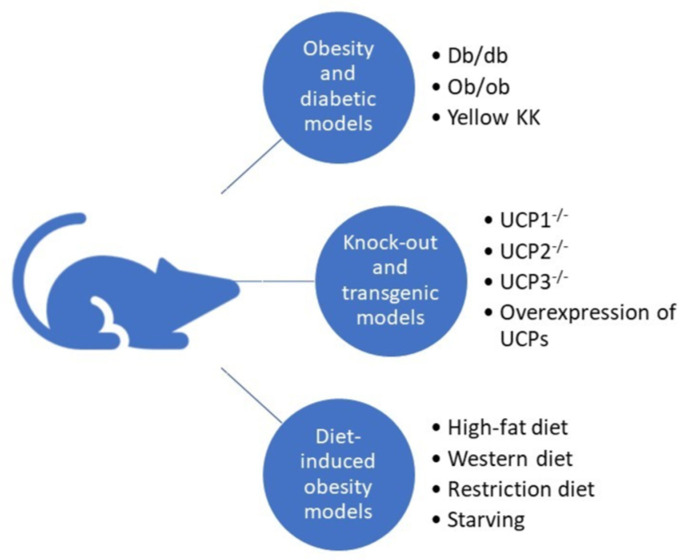
Different mouse models enable research on UCP’s roles in insulin sensitivity, diabetes, and obesity.

Obesity is an important factor affecting UCP expression in skeletal muscle. For this reason, there are many UCP expression studies using mouse models of obesity and diabetes (Figure 3). Masaki et al. studied the thermogenic roles of UCPs in different tissues in a cold-exposed db/db mouse model [147]. Cold is known to increase energy expenditure and thermogenesis [148]. Db/db mice resulted in impaired body temperature maintenance that presented as decreased thermogenic capacity. Compared to lean littermates, db/db mice were incapable of cold acclimation and had a diminished increase in UCP expression in skeletal muscle and brown and white adipose tissue. While cold exposure downregulated lipoprotein lipase mRNA and increased serum levels of FFAs in lean mice, these effects were not seen in db/db mice. These results suggest that in db/db mice, the reduced lipolysis may impair UCPs’ function [147].

In skeletal muscle, exposure to cold caused a reduction in UCP3 mRNA expression. The cause for the disruption of temperature maintenance in db/db mice is most likely a leptin-signaling malfunction, as db/db mice are leptin-receptor-mutated [149], thus confirming the vital role of leptin in regulating UCP expression. Further studies supported this idea by confirming increased UCP2 and UCP3 mRNA expression in pancreatic islets and muscle, respectively, in ob/ob mice with adenoviral-mediated leptin expression [150]. Leptin induces skeletal muscle UCP expression and sympathetic innervation [151], significantly contributing to thermogenesis. 

Apart from impaired leptin action in db/db mice, poor responsiveness of UCPs to regulation with serum FFAs has been suggested as a possible cause of thermogenesis impairment. FFAs are modulators of UCP regulation [152], and during cold exposure, their concentration in serum increases. Typically, this would trigger an increase in mitochondrial UCP3 expression in skeletal muscle as a mechanism to dispose of excess FFAs [153]. However, in db/db mice, acute elevation of FFAs in the serum after cold exposure failed to upregulate UCP3 mRNA expression in skeletal muscle [147].

In the last few decades, many studies searched for possible treatments to increase the amount of UCPs in skeletal muscle to achieve an antiobesity effect. Sympathetic nerves directly control skeletal muscle and adipose tissue thermogenesis through the β-adrenergic action of norepinephrine [154]. Nagase et al. (1996) used a β3-adrenergic agonist to increase UCP expression in muscles and BAT, which resulted in increased energy expenditure, enhanced oxygen consumption, improved glucose tolerance, and reduced body fat in obese yellow KK mice [155,156].

### 6.2. Transgenic and Knockout Mice

Besides the obesity and diabetic mouse models, knockout and transgenic mouse models enabled targeted studies of UCP roles [157] (Figure 3). UCP1 knockout mice can maintain their energy balance despite the absence of UCP1-mediated thermogenesis via higher proton-leak-dependent oxygen consumption in muscles [158]. In the adipose tissue–UCP1 knockout mice, the ectopic expression of UCP1 in skeletal muscle revealed an essential role of skeletal muscle respiratory uncoupling in preventing diet-induced obesity, insulin resistance, and cholesterolemia [159]. Accelerated metabolism in skeletal muscle by uncoupling activity due to UCP1 expression may also delay age-related diseases such as diabetes, hypertension, atherosclerosis, and cancer [160].

Furthermore, the roles of UCP1 in skeletal muscle and BAT are different. The impact of UCP1 on glutathione and ROS levels has been studied using a transgenic mouse model with selective expression of UCP1 in skeletal muscle. Even though UCP1 expression in skeletal muscle reached only up to 13% of levels compared to BAT, the increased GDP-sensitive proton leak through UCP1 in muscle cells increased total mitochondrial glutathione levels more than sevenfold compared to BAT [88]. However, unlike in BAT, the leak through UCP1 affected the mitochondrial ROS emission. Upon subsequent inhibition of UCP1 with GDP, ROS production in transgenic mice increased 2.8-fold relative to WT littermates [88], confirming the involvement of UCP1 in the mitochondrial oxidative state. 

UCP2 affects glucose homeostasis by controlling insulin secretion, food intake behavior, and adiponectin secretion in the pancreas, brain, and adipose tissue [161]. The role of UCP2 in β-cell glucose sensing was revealed in a study using ob/ob UCP2 knockout mice, as they expressed higher ATP levels and increased insulin secretion than the control group of ob/ob mice with an active *UCP2* gene [96]. Like UCP1 knockout mice, UCP2 knockout mice do not become obese when fed a HFD. Moreover, they have increased ROS production and a normal response to cold exposure [162]. Therefore, the negative regulation of insulin secretion by UCP2 represents a strong link between obesity, β-cell dysfunction, and the development of type 2 diabetes. On the other hand, overexpression of UCP2 proteins in mice decreases obesity and improves insulin sensitivity [163].

Furthermore, UCP3 promotes FFA oxidation in muscle, thereby indirectly influencing glucose metabolism [161]. Knockout mice lacking UCP3 have been created for physiological studies, and their phenotype was also confirmed to be nonobese. An increased ROS production and no significant thermoregulatory function of UCP3 were determined [108,164]. Transgenic mice expressing human ortholog of UCP3 at high levels in skeletal muscle were used in several studies and resulted in leanness. Their mitochondrial thioesterase-1 (MTE-1) and lipoprotein lipase expression were increased [153]. Adipose tissue mass decreased, and glucose clearance rate increased, making them resistant to obesity and diabetes development [163,165]. Within the mitochondria, MTE-1 cleaves acyl-CoA long-chain FFAs to FFA anions and CoASH. Further on, UCP3′s role is to export these anions from the mitochondrial matrix under conditions of increased β-oxidation [166,167]. Thus, UCP3 and MTE-1 have been implicated in FFA metabolism, and correlate with situations and tissues in which FFA β-oxidation is increased, such as skeletal muscle and BAT. 

### 6.3. Diet-Induced Obesity and Diabetic Models

Studies in various mouse models revealed expression and function changes of different UCPs in various tissues in response to specific diets (Figure 3). The Western diet, which is high in sugar, protein, and fat, and the HFD are often used to trigger the development of systemic insulin resistance, type 2 diabetes, and obesity. A study in UCP1 knockout mice showed that loss of UCP1 increased susceptibility to Western-diet-induced insulin resistance and glucose intolerance [168].

As the loci of UCP2 and UCP3 genes on mouse chromosome 7 and human chromosome 11 are close [107], they represent good candidate genes for the quantitative trait locus of diet-induced obesity and diabetes [169,170]. Research shows that diet can regulate UCP2 and UCP3 expression, and that their levels can increase due to fat consumption or refeeding after starvation [152,170]. In addition, cold stimulation and the sympathetic nervous system also regulate UCP3 content.

Feeding C57BL/6 mice with the HFD causes insulin resistance and reduced protein expression of GLUT4 and phosphorylation of AMPK in skeletal muscle and adipose tissue [171]. Interestingly, the HFD increases UCP2 expression in adipose tissue in obesity-resistant A/J mice, but not in obesity- and diabetes-prone mouse strains [170]. Similar results were obtained by Surwit et al. (1998). They also studied the correlation between the expression of different UCPs in adipose tissue and skeletal muscle, and the consumption of the HFD in obesity-prone C57BL/6J mice and obesity-resistant A/J and C57BL/KsJ mice. Their study indicated that the HFD increases UCP2 expression in white adipose tissue in obesity- and diabetes-resistant mouse strains. However, the HFD did not affect UCP2 expression in any tissue in obesity-prone mice. Moreover, the HFD did not affect UCP3 or UCP2 mRNA expression in skeletal muscle in any studied strain. Thus, the induction of diabetes and obesity by the HFD seems related mainly to UCP1 and UCP2 expression in adipose tissue, but not to the expression of UCP2 and UCP3 in skeletal muscle [172]. Therefore, uncoupling activity in adipocytes seems to have greater importance in controlling the effects of fat feeding on the development of obesity and diabetes than uncoupling activity in skeletal muscles. 

In mice with overexpression of a UCP, a more potent uncoupler than UCP2 or UCP3 in skeletal muscle, an enhanced insulin action and resistance to weight gain and insulin resistance induced by the HFD were determined [159]. Thus, skeletal muscle respiration uncoupling could contribute to treating obesity and pathologies linked to it. 

Many studies of UCP function used food restriction and fasting. A study in WT mice confirmed that fasting increases UCP2 and UCP3 expression in skeletal muscle while leaving proton leak unchanged [173]. Differences in respiratory quotients between wild-type and UCP3 knockout mice were found, and the absence of UCP3 resulted in impaired FFA oxidation. Altogether, these results suggest that UCP3 and UCP2 are highly linked to FFA oxidation and are therefore physiologically essential for FFA metabolism.

Moreover, mitochondrial dysfunction and spontaneous skeletal muscle apoptosis occur in muscle pathology mouse models such as collagen VI-knockout mice, which resemble human myopathy. Starvation and a low-protein diet proved beneficial for dystrophic muscles, as they protected them from atrophy and mitochondrial defects via induced autophagy [174] Not long ago, a low-protein/high-carbohydrate diet (LPCD) was shown to induce UCP1 expression, enhance mitochondrial oxidative metabolism, and recruit different energy-dissipating routes in murine beige adipocytes in subcutaneous adipose tissue. The induction of AMPK-dependent thermogenesis by the LPCD thus shows great potential as a valuable strategy for preventing metabolic diseases [175] The beneficial effects of caloric restriction have also been confirmed regarding muscle stem cells. The enhanced myogenic activity of satellite cells’ oxidative metabolism due to increased mitochondrial mass and function has been observed in mice that underwent short-term and long-term caloric restriction [176]. Therefore, caloric restriction has tremendous therapeutic potential in accelerating endogenous repair and improving the capacity of muscle stem cells. 

Apart from caloric restriction and fasting, short-term HFD has also been shown to counteract metabolic alterations in muscle fibers of dystrophic muscles. Fibro/adipogenic progenitors, which reside in muscle and represent an interstitial stem cell population of mesenchymal origin, have an important role in muscle regeneration [177]. Dystrophic progenitors have an impaired mitochondrial metabolism, which reduces their ability to proliferate and differentiate into adipocytes. Using dystrophic mice, HFD has been shown to metabolically reprogram these progenitors and modulate their adipogenic potential by restoring their mitochondrial functionality and UCP activity [178]. Several other studies confirmed the involvement of UCPs in muscle regeneration. It has been discovered that muscle fibro/adipogenic progenitors can differentiate into UCP1-expressing beige adipocytes, resulting in induction of muscle regeneration [179,180]. Moreover, transplantation of BAT into injured skeletal muscle has been shown to increase muscle mass and contractile force [181], pointing to a significant role of UCP1 in muscle regeneration and a great potential for therapy development.

The prevention of HFD consequences has been a hot topic in recent mice research. Polyphenols such as resveratrol, anthocyanin, curcumin, and epigallocatechin gallate have the potential to alleviate hyperglycemia and insulin sensitivity caused by the HFD. The most plausible mechanism of action involves stimulation of GLUT4 translocation on the plasma membrane of skeletal and cardiac muscle cells and adipocytes via an activated AMPK-dependent signaling pathway [171,182,183]. Besides GLUT4 translocation, the activation of AMPK regulates the expression of UCP3 in skeletal muscle [184,185]. Furthermore, polyphenol-rich cacao liquor extract has been successful as a supplement in preventing the development of hyperglycemia in the db/db obesity mouse model [186]. In addition, in C57BL/6 mice, supplementation of the HFD with cacao liquor extract prevented mice from diet-induced obesity. Polyphenols in cacao increased UCP3 expression in skeletal muscle and other UCP expressions in different tissues. This triggered the activation of AMPK, increased energy expenditure and thermogenesis, and prevented diet-induced hyperglycemia, insulin resistance, and obesity [171]. Metformin, notably an anti-diabetic drug, also has been extensively studied for its role in improving skeletal muscle metabolic and regenerative function. Metformin has several beneficial effects on stem cells, as it has been recently discovered and reported in many papers [187,188,189]. It is known to reduce ROS levels and protect mitochondria from oxidative damage, thus enabling an efficient activity of UCPs. Metformin is therefore developing as a valuable therapeutic for treating muscle atrophy and dystrophies, as studies by Pavlidou et al. demonstrated that metformin delayed satellite cell activation and differentiation by favoring a quiescent, low metabolic state, resulting in alleviated depletion of the stem cell pool and the functional loss of satellite cells [190].

### 6.4. Translational Precautions

Lately, the knowledge of mouse thermal physiology has raised questions about the relevance and translation of the results from preclinical studies on mice housed at a standard temperature to a clinical level due to the environmental temperature effects on energy homeostasis and metabolic rates [191]. As the body mass-to-surface ratio in mice is different from humans, the mouse body uses distinct thermo-biological processes to maintain temperature homeostasis [192]. In mice, an ambient temperature of about 23 °C triggers cold-induced thermogenesis devoted to maintaining core body temperature, mainly in BAT, representing more than one-third of the total energy expenditure. The basal metabolic rate, physical activity, and the thermogenic effect of food account for the remaining energy expenditure. To circumvent this energy-consuming process, mice can enter regulated hypothermia [193], which does not even closely resemble human sedentary processes at this temperature [194]. This fact has led scientists to conclude that thermoneutral points for humans and mice are far from similar and must be considered when conducting studies of obesity and diabetes mechanisms and treatment in mouse models. In further studies, researchers determined that the thermoneutral point for mice is around 30 °C, with energy expenditure consisting of approximately 70% basal metabolic rate, 20% physical activity energy expenditure, and 10% thermic effect of food [195]. Therefore, this environmental temperature should be used to better model human energy homeostasis in mouse models [196].

## 7. Conclusions

Maintaining temperature homeostasis is mainly achieved through thermogenic processes involving UCPs in BAT and skeletal muscle. The same proteins are involved in glucose homeostasis, thus linking high-energy dissipation and body weight control, a promising research topic for treating obesity and type 2 diabetes. Research in obesity and diabetic mouse models has demonstrated that leptin and serum levels of FFAs play an essential role in regulating UCP3 expression in skeletal muscle, and that muscle thermogenesis in these models is impaired. To elucidate the specific roles of different UCPs, knockout and transgenic mouse models have been created. Experimental data revealed that overexpressing UCP1 in skeletal muscle can accelerate metabolic energy consumption and prevent diet-induced obesity and insulin resistance. On the other hand, UCP2 overexpression decreases insulin secretion in beta-cells, leading to obesity, β-cell dysfunction, and type 2 diabetes. Changes in UCP expression in different tissues can also result from high-fat and/or high-carbohydrate diets such as the Western diet. However, such diets mainly affect UCP expression in adipose tissue, but have an insignificant impact on the skeletal muscle. 

Harnessing the processes of thermogenic systems based on the UCPs offers a great potential to reduce obesity and diabetes. As many UCPs homologs are expressed in a broad range of tissues, many targets exist to induce mitochondrial uncoupling to stimulate energy expenditure. In future research, attention should be paid to the thermoneutral point and environmental temperature when conducting studies in mice for a better translation of findings from mouse models to humans.
